# Detection of Bacterial Pathogens from Broncho-Alveolar Lavage by Next-Generation Sequencing

**DOI:** 10.3390/ijms18092011

**Published:** 2017-09-20

**Authors:** Stefano Leo, Nadia Gaïa, Etienne Ruppé, Stephane Emonet, Myriam Girard, Vladimir Lazarevic, Jacques Schrenzel

**Affiliations:** 1 Genomic Research Laboratory, Service of Infectious Diseases, Geneva University Hospitals (HUG), 1205 Geneva, Switzerland; nadia.gaia@genomic.ch (N.G.); etienne.ruppe@genomic.ch (E.R.); myriam.girard@genomic.ch (M.G.); vladimir.lazarevic@genomic.ch (V.L.); jacques.schrenzel@hcuge.ch (J.S.); 2 Service of Infectious Diseases, Geneva University Hospitals and Faculty of Medicine, 1205 Geneva, Switzerland; stephane.emonet@hcuge.ch; 3 Bacteriology Laboratory, Geneva University Hospitals and Faculty of Medicine, 1205 Geneva, Switzerland

**Keywords:** whole-metagenome shotgun, clinical metagenomics, broncho-alveolar lavage

## Abstract

The applications of whole-metagenome shotgun sequencing (WMGS) in routine clinical analysis are still limited. A combination of a DNA extraction procedure, sequencing, and bioinformatics tools is essential for the removal of human DNA and for improving bacterial species identification in a timely manner. We tackled these issues with a broncho-alveolar lavage (BAL) sample from an immunocompromised patient who had developed severe chronic pneumonia. We extracted DNA from the BAL sample with protocols based either on sequential lysis of human and bacterial cells or on the mechanical disruption of all cells. Metagenomic libraries were sequenced on Illumina HiSeq platforms. Microbial community composition was determined by k-mer analysis or by mapping to taxonomic markers. Results were compared to those obtained by conventional clinical culture and molecular methods. Compared to mechanical cell disruption, a sequential lysis protocol resulted in a significantly increased proportion of bacterial DNA over human DNA and higher sequence coverage of *Mycobacterium abscessus*, *Corynebacterium jeikeium* and *Rothia dentocariosa*, the bacteria reported by clinical microbiology tests. In addition, we identified anaerobic bacteria not searched for by the clinical laboratory. Our results further support the implementation of WMGS in clinical routine diagnosis for bacterial identification.

## 1. Introduction

Sample culturing is the main method used to identify pathogens in clinical microbiology. However, it requires bacterial growth on different media as well as various atmospheric and temperature adjustments. Compared to laboratory procedures, culture-independent screening conducted with whole-metagenome shotgun (WMGS) only needs a small amount of DNA directly taken from the sample and a bioinformatics tool which identifies bacteria by linking sequencing reads to a curated reference genome (or marker) database. Moreover, the availability of whole-genome sequences and taxonomic markers from microorganisms, including yet-uncultivable ones, makes it possible, with metagenomics, to provide a more comprehensive overview of the whole microbiota. Eventually, if the sequence depth is sufficient enough, the genome of a given bacterium could be recovered and antibiotic susceptibility could be inferred. WMGS can be applied to different types of samples obtained by non-invasive (e.g., urine [1,2,3]) or invasive (e.g., bone [4]) techniques.

Despite some advantages, WMGS is not routinely used in clinical microbiology. Few clinical single-patient studies investigated with metagenomics have been reported in the literature and they mostly concern viral acute encephalitis [5], infective endocarditis [6], or bacterial meningitis [7,8]. There are several reasons why WMGS has not yet been extensively used in routine analyses. First, in some types of clinical samples a high proportion of human DNA fragments relative to bacterial DNA fragments in DNA extracts and in metagenomic libraries reduces the sensitivity of WMGS in identifying bacteria. Second, the cost of WMGS is not affordable for all public hospitals and, third, the analyses of sequencing data generated require substantial computational skills and resources. Therefore, clinical metagenomics has not yet proved its benefits over conventional methods.

In the last few years, several tools have been designed specifically for metagenomic data analyses. Some, like Metagenomic Phylogenetic Analysis version 2 (MetaPhlAn2) [9], make use of gene catalogues containing taxon-specific markers against which query reads are aligned. Others, like CLAssifier based on Reduced K-mers (CLARK) [10] and Kraken [11], decompose both reference and query sequences in k-mers of k-length and reduce the computational speed by using exact-match alignment criteria.

Here we present and discuss a metagenomic approach that challenges the problem of human DNA contamination and makes use of relatively memory-saving bioinformatics approaches. In particular, we applied such a pipeline on a broncho-alveolar lavage (BAL) sample collected from an immunocompromised patient diagnosed with severe chronic pneumonia. As metagenomic libraries prepared from respiratory tract samples may contain relatively high amount of human DNA fragments [12], we applied two different DNA isolation procedures by including (or not) the selective enrichment of bacterial/fungal DNA. After WMGS we computed the relative abundance of reads mapping to human, fungal, viral and bacterial species with the read classification tools CLARK and Kraken. The identification of bacteria was also performed by a search for taxonomic markers with MetaPhlAn2. We analyzed the abundance of genes involved in antibiotic resistance and attempted to link them to the corresponding genomes. Finally, the results obtained by metagenomics were compared with clinical microbiology records.

## 2. Results and Discussion

### 2.1. Pre-Processing of Sequencing Data

**Figure 1:**
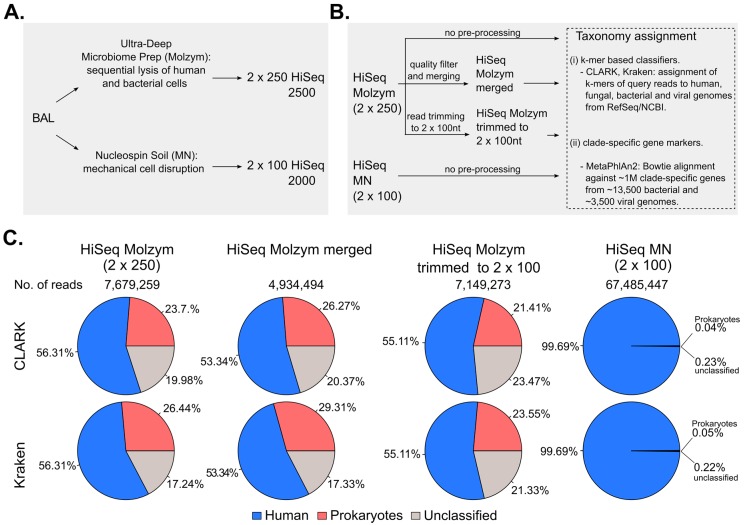
(**A**) Schematic representation of metagenomic sequencing: BAL was split into two aliquots which were independently treated with two DNA extraction protocols: Ultra-Deep Microbiome Prep (Molzym) and NucleoSpin Soil (MN). Metagenomic libraries were independently sequenced with 2 × 250 HiSeq 2500 and 2 × 100 Hiseq 2000, respectively; (**B**) Schematic representation of bioinformatics analyses: forward and reverse raw reads of Molzym- (HiSeq Molzym (2 × 250)) and MN-treated (HiSeq MN (2 × 100)) samples were used for taxonomic analysis with CLARK, Kraken and MetaPhlAn2. In addition, before taxonomic analyses, read pairs of the HiSeq Molzym (2 × 250) dataset were either quality-filtered and merged (HiSeq Molzym merged) or trimmed to the length of 100 nt (HiSeq Molzym trimmed to 2 × 100) as described in Materials and Methods; (**C**) Pie-charts representing the proportions (%) of sequencing reads classified as human (in blue), prokaryotic (red) or unclassified (grey) by CLARK (top row) and Kraken (bottom row). Proportions were computed over the total number of reads present in a given dataset. Viral- and fungal-classified read proportions are not shown and contributed to less 0.02% of total reads in all sequencing datasets.

Paired-ended sequencing of DNA preparations obtained without (NucleoSpin Soil kit, Macherey-Nagel, Düren, Germany) and with bacterial/fungal DNA enrichment (Ultra-Deep Microbiome Prep kit, Molzym, Bremen, Germany) was performed by Illumina HiSeq 2000 and by Illumina HiSeq 2500 platforms (Figure 1A), respectively. HiSeq sequencing of the unenriched sample, containing lower proportions of bacterial DNA, allowed high sequencing depth (67,485,447 read pairs) in a cost-efficient manner, though at the expense of the read length (100 nt versus 250 nt for enriched samples).

To facilitate the comparison of the results, we also trimmed 250-nt reads of the enriched sample to 100 nt (Figure 1B) which resulted in a dataset containing 93.1% of initial raw reads, that is 7,149,273 sequence pairs. The observed reduction in read number was due to the concomitant removal of reads <100 nt.

As raw sequencing reads had median quality scores reflecting low sequencing error probability across all positions (<0.001) (Supplementary Figure S1), sequencing datasets were directly processed by CLARK and Kraken, without applying any quality filter or pair merging steps. Using the enriched sample dataset, we also assessed the effect of the removal of low quality reads and merging of paired reads on species identification (Figure 1B). After read pairing, the resulting dataset contained 64.3% (4,934,494) of the initial number of read pairs (7,679,259).

### 2.2. Effect of Host DNA Depletion on the Proportions of Bacterial Reads

The extract obtained by mechanical cell disruption without microbial DNA enrichment contained high amounts of human DNA as shown by both qPCR assay (99.88% of total DNA) and shotgun sequencing (99% of total reads) (Table 1; Figure 1C). qPCR analysis showed that microbial enrichment resulted in about 230-fold and 310-fold increase in the proportion of bacterial DNA and in the bacterial/human DNA ratio, respectively (Table 1). Consistently, the percentage of reads mapped to bacterial genomes by CLARK and Kraken in datasets from enriched samples reached 23.7% and 26.44%, while being <0.1% in unenriched sample dataset (Figure 1C). Merging and quality filtering of the read dataset from the enriched sample resulted in a 3% higher proportion of reads classified to prokaryotes (Figure 1C). On the other hand, trimming the reads of the enriched sample from 250 to 100 nt length decreased the proportion of prokaryotic reads by 3% (Figure 1C).

### 2.3. Identification of Bacterial Species by Metagenomics

**Figure 2:**
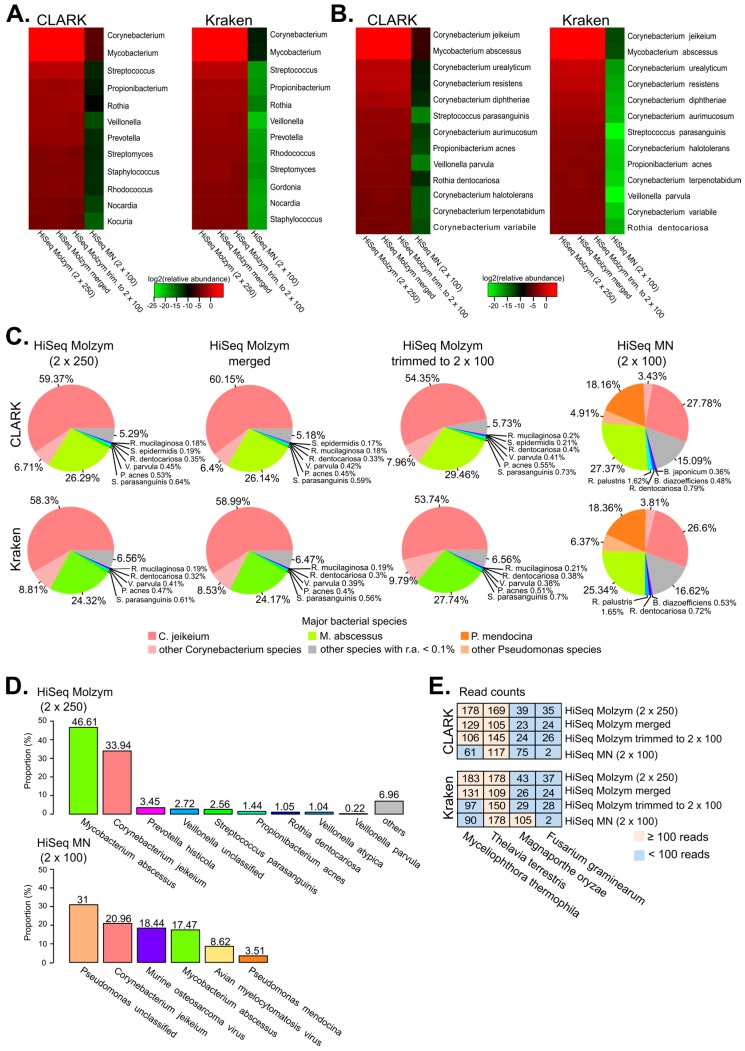
Heat maps reporting the log2-transformed relative abundance of the 15 most abundant bacterial genera (**A**) and the 16 most abundant bacterial species (**B**) identified by CLARK and Kraken in all sequencing datasets. Relative abundance, expressed in percentage, is computed on the number of reads assigned to a given genus or species divided by the total number of sequencing reads present in a given dataset; (**C**) Pie charts representing the bacterial proportions of identified species as performed by CLARK (top) and Kraken (bottom). Bacterial proportions are defined as the percentage of reads mapped to a given bacterial species over the total number of reads assigned to prokaryotes; (**D**) Barplots showing relative abundance (%) of bacterial and viral taxa identified by MetaPhlAn2 in HiSeq Molzym, and HiSeq MN sequencing data. Percentage values are reported at the top of each bar; (**E**) Heat map representing the number of reads assigned to the most abundant fungi. Numbers indicate read counts instead of proportions.

Metagenomic analyses performed by CLARK and Kraken on non-preprocessed and preprocessed sequencing data of the enriched BAL aliquot identified *Corynebacterium*, *Mycobacterium*, *Streptococcus*, *Propionibacterium*, *Rothia* and *Veillonella* among the top six most abundant genera (Figure 2A). Analyses at the species level detected some *Corynebacterium* species dominated by *Corynebacterium jeikeium*, also found by culture (Figure 2B; Table 2). Sequencing data also confirmed laboratory results i.e., the presence of two other bacteria, *Mycobacterium abscessus* and *Rothia dentocariosa* (Figure 2B; Table 2). 

Moreover, CLARK- and Kraken-based analyses identified three species not detected by culture (Figure 2B): *Streptococcus parasanguinis* and *Veillonella parvula*, which are known colonizers of human oral cavity [13], and *Propionibacterium acnes*. The possible reasons for not detecting these bacteria in culture are that: *V. parvula* is an anaerobic bacterium, *S. parasanguinis* is not necessarily reported by clinical laboratory if present in a subdominant proportion, and *P. acnes* is a known reagent contaminant [14].

Among the bacteria identified by CLARK and Kraken, *C. jeikeium* and *M. abscessus* were dominant over other species (Figure 2C). A fraction of bacterial reads (in the range between 3.4% and 9.8%) was assigned to other *Corynebacterium* species, probably reflecting misclassification errors. Other identified species, including *R. dentocariosa*, *S. parasanguinis*, *P. acnes* and *V. parvula* were each represented by less than 1% of the reads assigned to bacteria. 

Mapping sequencing reads from the enriched sample to clade-specific markers using MetaPhlAn2, identified *M. abscessus* as prominent species, followed by *C. jeikeium* (Figure 2D), in contrast to results of CLARK- and Kraken-based analyses, where *C. jeikeium* was more abundant than *M. abscessus*. This difference was not directly related to the differences in genome size between the two species, since the *M. abscessus* genome is almost twice as that of *C. jeikeium* (5 Mbp versus 2.5 Mbp [15,16]) and MetaPhlAn2, in contrast to CLARK and Kraken, computes species relative abundance by normalizing reads according to marker gene length. 

Although quality filtering would be a recommended step in the metagenomic analysis of clinical samples, trimming and quality filtering/merging of sequence data in the present study did not change the taxonomic profile of the enriched BAL sample much: the species identified by culture (*C. jeikeium*, *M. abscessus* and *R. dentocariosa*) were among the most abundant bacteria in both non-preprocessed and preprocessed datasets (Figure 2B,C).

The proportion of reads assigned to *C. jeikeium* in the bacterial component of the metagenome of the non-enriched sample was also reduced in comparison to the enriched sample (Figure 2C). In contrast, the *Pseudomonas* species, not observed in the culture analysis and poorly represented in the enriched dataset (<0.2% of bacterial proportions), corresponded to >20% of reads assigned to bacteria (Figure 2C). Although we cannot exclude that some bias was introduced by the DNA extraction method, the reads assigned to *Pseudomonas* most likely originated from reagent contaminants as shown in a previous study [17]. 

Other putative reagent contaminants in the unenriched sample included members of Bradyrhizobiaceae family: *Rhodopseudomonas palustris*, *Bradyrhizobium diazoefficiens* and *Bradyrhizobium japonicum* (Figure 2C). A clearer distinction between reagent contaminants and genuine microbiota would require the sequencing of negative extraction controls. 

### 2.4. Detection of Fungi and Viruses

Consistent with the results on the bacterial component of the microbiome, the fraction of reads assigned to most abundant viruses and fungi were not affected much by read pre-processing (Figure 2E; Table 3). However, the impact of DNA extraction method on the relative abundance of fungal and viral reads (expressed as percentage of all reads) was marked. The read proportion of the most abundant fungi increased 10- and 15-fold in the enriched dataset with Kraken and CLARK, respectively, compared to non-enriched BAL (Figure 2E). 

In contrast, the enrichment procedure resulted in about a 6-fold reduction of the proportion of reads assigned to herpes viruses (Table 3). Torque teno viruses were dominant over the other viral species in all sequencing datasets (Table 3). To increase the potential of WMGS to capture viral, bacterial and fungal pathogens, clinical samples should be processed in parallel by different methods. Techniques for the selective enrichment of viral DNA/RNA prior to metagenomic sequencing have been recently developed [18]. 

About 0.04% of sequencing reads were assigned to the Illumina library control phage phiX174 in the HiSeq 2000 sequencing run of the unenriched sample, while no such reads were identified in the HiSeq 2500 dataset (enriched sample). 

In datasets of enriched sample, some reads mapped to phages of the genera *Propionibacterium*, *Mycobacterium*, *Streptococcus* and *Staphylococcus* (Table 3). These phages were identified at a lower proportion or not at all in the unenriched sample dataset. It seems likely that the reads mapping to these specific phages originated from prophages rather than free phage particles which are expected to be removed by the microbial enrichment procedure. 

*Purpureocillium lilacinum*, reported in culture analysis, was not specifically detected by metagenomics because of the lack of its genomic sequence in the RefSeq/NCBI reference genome database used for the present analysis. The four most abundant fungi identified by CLARK and Kraken (*Fusarium graminearum*, *Magnaporthe oryzae*, *Thelavia terrestis and Myceliophthora thermophila*) are species of agricultural rather than clinical interest. However, they belong to the class *Sordariomycetes*, as is the case of *P. lilacinum*. 

Accurate detection of fungal (and other) species by CLARK and Kraken is critically dependent on the number of available reference genome sequences. Successful detection of fungal species would also require more genomes to be sequenced.

### 2.5. Analysis of Antibiotic Resistance Determinants (ARDs)

**Figure 3:**
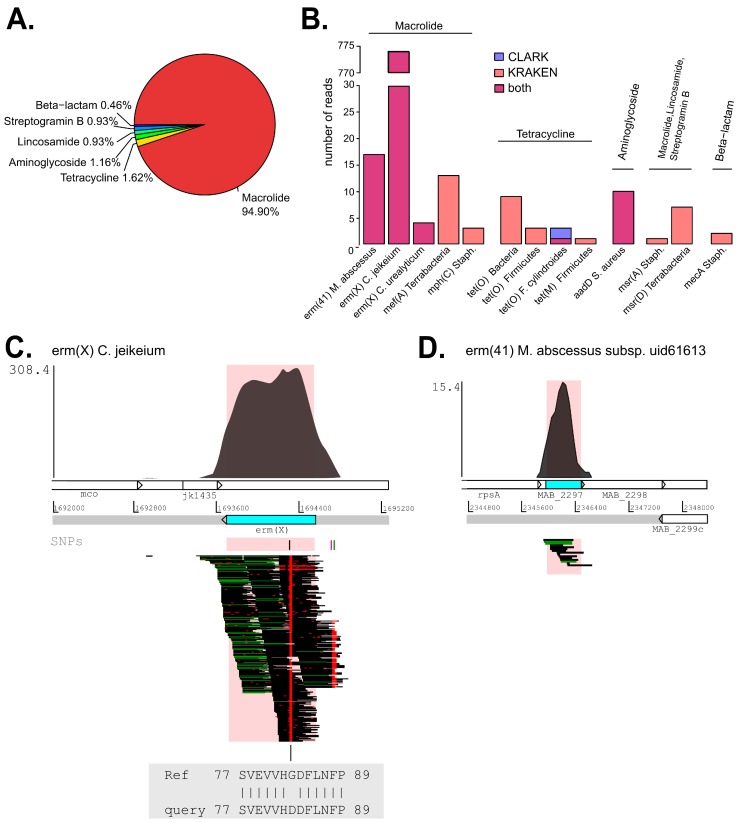
(**A**) Pie chart representing the relative abundance of the antibiotic resistance-determinant (ARD) classes in the metagenome of the enriched BAL sample after quality filtering and merging of sequencing reads. Relative abundance is expressed as percentage of total reads mapped to the ARD ResFinder database (847); (**B**) Barplot reporting the number of reads mapping to the identified ARDs. Colors indicate whether the taxonomic read assignment of an ARD to a given taxon was performed with CLARK (blue violet), with Kraken (coral) or confirmed by both (magenta). Reads that mapped to *cfxA* and *cfxA2* genes remained unclassified; (**C**) Coverage plot of reads mapping to *erm*(X) locus on the *C. jeikeium* K411 genome. From top to bottom: density plot of read depth versus genome position; chromosomal position of the *erm(X)* gene; the open reading frame is colored in sky blue; position of three main single-nucleotide polymorphisms (SNPs) detected; schematic representation of mapped reads grouped in stacks; red dots and lines on stacks represent a nucleotide variant in a given read sequence; amino acid substitution in the ERM(X) protein. The pink-colored area defines the chromosomal landmarks of the gene in the density plot and in the representation of read mapping; (**D**) Coverage plot of reads mapping to the *erm*(41)-MAB_2297 locus on the *M. abscessus* ATCC 19977 genome. For further explanation, see panel (**C**). No single nucleotide variants were detected.

We eventually looked for genes that are involved in antibiotic resistance to link, if possible, antibiotic susceptibility testing (AST) for *M. abscessus* and *C. jeikeium* performed by the bacteriology laboratory (Supplementary Table S1) to our metagenomic data. Therefore, we first identified ARDs present in the enriched BAL metagenome by mapping quality-filtered and merged reads against the ResFinder ARD database [19]. In total, 847 reads were aligned to ResFinder database, most of which (94.9%) mapped to genes associated with resistance to macrolide antibiotics (Figure 3A; Table 4).

We then investigated whether those ARDs came from *M. abscessus* and *C. jeikeium* either by mapping reads against reference genomes with USEARCH [20] or by retrospectively looking to the taxonomic read assignments performed by CLARK and Kraken. 

Only two ARDs, *erm*(X) and *erm*(41), were classified to *M. abscessus* and *C. jeikeium*: 774 out of 778 reads mapping to *erm*(X) were classified to *C. jeikeium*, whereas all the 17 reads mapping to *erm*(41) corresponded to *M. abscessus* (Table 4; Figure 3B). None of the other ARD genes were associated to any of bacteria as detected by culture nor to the most abundant species as detected by metagenomics.

To determine whether *erm*(X) and *erm*(41) are chromosomal or plasmid-borne, we mapped corresponding reads against the reference genomes with BWA [21]. ARDs were considered chromosomal if BWA-aligned reads covered the neighboring regions of the gene on the bacterial chromosome, as was the case for both *erm*(X) and *erm*(41) (Figure 3C,D).

For *erm*(X), we found a single nucleotide difference from the reference sequence (GGT to GAT) in all relevant reads (Figure 3C). This change results in a glycine-to-aspartic acid substitution at position 83 of ERM(X) protein (Figure 3C). A BLASTP search carried out at the NCBI website revealed that ERM(X)-Asp83 is a variant present within other species of the phylum of Actinobacteria. However, no information is reported in literature concerning the contribution of ERM(X)-Asp83 to the acquisition, promotion or inhibition of antibiotic resistance. Compared to *erm*(X), *erm*(41) of *M. abscessus* was poorly covered and no single-nucleotide variants were found in corresponding sequencing reads (Figure 3D).

*erm*(X), the most representative ARD in our metagenomics analysis, has been described as promoter of resistance to lincosamides and macrolides in other Actinobacteria species [22] and it could explain why *C. jeikeium* was reported as resistant to clindamycin by routine AST. On the other hand, *M. abscessus* identified by culture was phenotypically susceptible to macrolides, despite the detection of *erm*(41) with no nucleotide substitutions relative to the reference ResFinder sequence. Although for both bacteria identified, sequencing read depth was more than 20× (Supplementary Table S2), it was difficult to correlate the phenotypic antibiotic resistance with metagenomic data. A possible explanation is that ARDs in the identified strains might differ from those present in ResFinder database. In addition, our bioinformatics approach does not detect mutations conferring antibiotic resistance. 

## 3. Materials and Methods

### 3.1. Patient Description

The patient reported here was a 38-year-old man who had benefited from allogenic human stem cell transplantation (HSCT) for lymphoblastic leukemia. He developed severe graft-versus-host disease (GvHD) involving the digestive, hepatic and pulmonary tracts, together with other complications like human herpesvirus 6 (HHV6) encephalitis, cytomegalovirus reactivation and thrombotic microangiopathy. Because of the GvHD, major immunosuppressive treatment was administered. Six months after the HSCT, he developed a left lobar pneumonia. Empiric treatment with levofloxacin for 10 days did not attenuate neither the symptoms (cough and fever) nor the lung damage (excavation of the lung lesion). Cultures from BAL sample revealed the presence of *M. abscessus* and two fungi, *Scopulariopsis* spp. and *P. lilacinum* (Table 2). *C. jeikeium* and *R. dentocariosa* were also reported in the cultures (Table 2) but they were considered as colonizers in this situation. Despite a combined antifungal treatment (liposomal amphotericin B and posaconazole) and triple-therapy against *M. abscessus* (clarithromycin, imipenem, ciprofloxacin), the patient developed respiratory distress, leading to his death. The publication and dissemination of the analyses were authorized by the Cantonal Ethics Committee of Geneva on 16 May 2017 without the need to submit a request to the ethics committee in accordance with the legislation of Geneva canton since the study is a single-patient case report.

### 3.2. Culture Methods and Antibiograms

A 5-mL aliquot of the BAL fluid was homogenized by shaking and centrifuged. The pellet was suspended (0.5 mL), inoculated in conventional fungal medium [23], and incubated at 37 °C ± 1 for 14 days. Another aliquot was used for Gram and fungifluor staining, and immediate inoculation onto Columbia blood agar, MacConkey agar, Columbia colistin-nalidixic acid (C. N. A.) agar, chocolate agar, and brain–heart broth. The solid media and brain–heart broth were incubated in a CO_2_-enriched atmosphere for 4 days at 37 °C ± 1. The identification of the *C. jeikeium* and *R. dentocariosa* isolates was performed using matrix-assisted laser desorption/ionization time-of-flight mass spectrometry (MALDI-TOF MS; Maldi Biotyper 2.0, Bruker Daltonics, Bremen, Germany) according to the manufacturer’s instructions. Acid-fast bacilli cultures were processed by using the BBL MGIT PANTA Antibiotic Mixture (Becton Dickinson), in addition to Stonebrink and Coletsos media (incubated for 14 weeks at 36.5 °C). The identification of acid-fast bacilli and fungal pathogens was carried out by PCR and sequencing as described previously [24,25]. All antibiotic susceptibility testing (AST) was performed according to the European Committee on Antimicrobial Susceptibility Testing (EUCAST) guidelines (edited in 2014; Available online: http://www.eucast.org/). 

### 3.3. DNA Extraction without Selective Bacterial DNA Enrichment and Sequencing 

DNA was extracted from 600 μL of BAL sample using the NucleoSpin Soil kit (Macherey-Nagel, Düren, Germany) as described previously [17] except that bead beating was performed for 20 min. DNA was eluted twice with 2 × 30 μL of elution buffer SE of the NucleoSpin Soil kit. Purified DNA was stored at −20 °C. DNA concentration was measured using the Qubit fluorometer with Qubit dsDNA HS Assay Kit (Life Technologies, Carlsbad, CA, USA) as recommended by the manufacturer. Paired-end metagenomic DNA libraries were prepared from 100 ng DNA using the Ovation Rapid Multiplex DR System 1–96 (Nugen, San Carlos, CA, USA) and size-selected at about 300–400 bp (including adapters). The libraries were sequenced on an Illumina HiSeq 2000 (Illumina, San Diego, CA, USA) using the 2 × 100 base paired end method at LGC Genomics (Berlin, Germany). Demultiplexed fastq files were generated from base-calls using Illumina’s bcl2fastq v1.8.4 software (Illumina, San Diego, CA, USA). Clipping of sequencing adapter remnants was performed using proprietary LGC Genomics software (LGC, Teddington, Middlesex, UK).

### 3.4. Enrichment of Bacterial/Fungal DNA and Sequencing

DNA was extracted from 600-μL BAL sample using Ultra-Deep Microbiome Prep (Molzym, Bremen, Germany) according to the manufacturer’s instructions (Version 2.0) for liquid samples. Fifty μL of DNA extract, containing 1.85 ng of DNA (the sum of bacterial and human DNA determined by qPCR) was further purified at Fasteris (Plan-les-Ouates, Geneva, Switzerland) by a proprietary procedure and eluted in 10 μL. Paired-end metagenomic DNA libraries were prepared from 5 μL eluate with the Nextera XT DNA Sample Preparation Kit, according to the Illumina (San Diego, CA, USA) using 16 (instead of 12) PCR enrichment cycles and 3 instead of 5 μL Nextera tagmentase. The libraries were sequenced in Rapid Run mode for 2 × 250 + 8 cycles on an Illumina HiSeq 2500 instrument at Fasteris (Geneva, Switzerland) using HiSeq Control Software 2.2.58 (Illumina, San Diego, CA, USA). Demultiplexed fastq files were generated with CASAVA-1.8.2 (Illumina, San Diego, CA, USA) from on-instrument base-calling by Real-Time Analysis (RTA) software 1.18.64.0 (Illumina, San Diego, CA, USA). The Trimmomatic package [26] was used by the sequencing service provider to remove bases corresponding to the standard Illumina adapters and to trim the ends of sequencing reads when the average Phred quality of a 4-base sliding window was less than 5.

### 3.5. qPCR Assays

The concentration of bacterial and human DNA was determined by qPCR experiments targeting the 16S rRNA and human β-actin genes, respectively, as described previously [17]. The reference curves for bacterial and human DNA quantitation were generated using known concentrations of *Escherichia coli* DH5α genomic DNA and human genomic DNA from the Applied Biosystems TaqMan β-Actin Detection Reagent kit (Thermo Fisher Scientific, Carlsbad, California, USA), respectively.

### 3.6. Bioinformatics Analyses

Bioinformatics analyses were run on a Unix workstation with 280 GB of RAM. Raw reads were quality checked by FastQC (Available online: http://www.bioinformatics.babraham.ac.uk/projects/fastqc/). Fastq files were directly used for taxonomic read assignments with the two k-mer based classifiers CLARK (full mode) and Kraken with default k-mer length of 31. For CLARK, we used a confidence score of read assignment of 0.75. Identification of bacterial species was also performed with MetaPhlAn2 with default settings. In addition, paired reads derived from Molzym-extracted DNA were quality filtered and merged with Paired-End reAd mergeR (PEAR) [27] with the following settings: maximum assembly length (-m) = 575; minimum assembly length (-n) = 200; minimum overlap (-v) = 25; minimum read size after trimming (-t) = 120; *p*-value (-*p*) = 0.0001; maximal proportion of uncalled bases (-u) = 0; and quality score threshold in trimming (-q) = 33. Read trimming to 100 nt in length of the fastq files of enriched sample was performed with USEARCH v7; reads that were shorter than 100 nt were removed. For the identification of antibiotic resistance genes, we used the quality-filtered and merged reads from the Molzym-treated BAL sample. Reads were mapped to the ResFinder database with UBLAST [20] with the following settings: identity (-id) = 0.9; *e*-value threshold (-evalue) = 0.00001; finding hits on both the forward and reverse strands (-strand both); keeping hits with the best identity or best *e*-value scores (-top_hits_only); and minimum length of the alignment (-mincols) = 125. For sequence reads with multiple best hits, we kept the hit corresponding to the resistance gene that was the most frequently assigned in our analysis. ResFinder-mapping reads were aligned against *C. jeikeium* K411 and *M. abscessus* ATCC 19977 reference genomes with USEARCH by considering three cutoffs for sequence identity: 30%, 50% and 90%. Reads mapping to *erm*(X) and *erm*(41) genes were taxonomically classified with CLARK and Kraken as explained above and eventually mapped against the corresponding genomes with the Burrows–Wheeler Aligner BWA-MEM v0.7.15-r1140. BAM files were generated, sorted and indexed with SAMtools software package [28] v1.3.1. Single nucleotide variant analyses were performed with bcftools, implemented in SAMtools. Artemis [29] was used for graphical representation, statistics of the alignments and SNP localization. R v3.2.3 was used for production of plots and graphs.

### 3.7. Genome Databases

Human (GRCh38), 34 fungal, 2785 bacterial and 4371 viral reference genome sequences were downloaded from RefSeq/NCBI with an adapted version of the CLARK command script “download_data.sh”. 

### 3.8. Data Availability

Adapter-clipped sequences were deposited as fastq (R1 and R2) files at the European Nucleotide Archive (ENA) under the project PRJEB20877 after removal of human sequences by screening against the *Homo sapiens* genome (GRCh38) using Kraken. 

## 4. Conclusions

We showed that partial removal of host DNA from a BAL sample using the Molzym Ultra-Deep Microbiome Prep isolation protocol increases detection sensitivity of bacterial and fungal species while decreasing that for viruses. The bioinformatics pipeline we used was sensitive enough to detect bacterial species found by culture and molecular tests in the clinical diagnostic laboratory. In addition, it allowed for identification of microorganisms that were not searched for in the routine tests, such as anaerobic bacteria and viruses. Although non-preprocessed and preprocessed datasets produced similar taxonomic profiles, a quality filtering step would be recommended before taxonomic assignments with CLARK and Kraken, notably when the sequencing data are of lower quality. We managed to identify some ARD genes and link them back to corresponding genomes. WMGS, although not yet standardized for clinical applications, is complementary with routine diagnostic tests based on culture and molecular approaches for bacterial identification, providing deeper insights in the composition of the microbiota in clinical samples. 

## Figures and Tables

**Table 1:** Bacterial and human DNA yields measured by qPCR.

DNA Extraction Kit	DNA Concentration (pg/μL) in the Extracts Estimated by qPCR		Input Sample Volume (μL)	DNA Extract Volume (μL)	Percentage of Bacterial DNA in Extracts	Percentage of Human DNA in Extracts	Ratio Bacterial-Human DNA
	Human	Bacterial					
Nucleospin Soil-MN	4197	5	600	60	0.12	99.88	0.0012
Ultra-Deep Microbiome Prep-Molzym	27	10	600	100	27.03	72.97	0.37

**Table 2:** Culture and molecular data from the bacteriology laboratory.

**Bacteria**	**Detection**
*Corynebacterium jeikeium*	>1.0 × 10^5^ CFU/mL
*Rothia dentocariosa*	1.0 × 10^2^ CFU/mL
*Mycobacterium abscessus*	Present
**Fungi**	**Detection**
*Pupureocillium lilacinum*	Present
*Penicillium* sp.	Present
*Scopulariopsis* sp.	Present

CFU, colony forming units.

**Table 3:** Read counts per sample of most abundant viruses.

	CLARK				Kraken			
Viruses	HiSeq Molzym (2 × 250)	HiSeq Merged	HiSeq Trim. to 100 nt	HiSeq MN (2 × 100)	HiSeq Molzym (2 × 250)	HiSeq Merged	HiSeq Trim. to 100 nt	HiSeq MN (2 × 100)
Torque teno viruses	198	119	188	2313	298	208	248	2992
*Propionibacterium* phage	35	15	33	3	50	26	45	3
*Mycobacterium* phage	26	12	17	0	27	12	17	0
*Staphylococcus* phage	26	12	23	0	24	17	12	0
*Streptococcus* phage	24	17	15	0	29	15	23	0
Enterobacteria phage phiX174	0	0	0	27,393	0	0	0	27,405
Human herpesvirus	4	1	2	193	4	1	2	230
Falconid herpesvirus	0	0	0	133	0	0	0	144

**Table 4:** Number of sequencing reads mapped to ResFinder database and genomic reference sequences of *C. jeikeium* and *M. abscessus*.

			ResFinder	*C. jeikeium*			*M. abscessus*		
		Identity:	0.9	0.3	0.5	0.9	0.3	0.5	0.9
Gene	Allele	Classes of Antibiotics							
*erm*(X)	*erm*(X)_4_NC_005206	Macrolide	544	544	544	538	0	0	0
*erm*(X)	*erm*(X)_2_X51472	Macrolide	185	185	185	181	0	0	0
*erm*(X)	*erm*(X)_1_M36726	Macrolide	49	49	49	49	0	0	0
*erm*(41)	*erm*(41)_1_EU177504	Macrolide	17	0	0	0	17	17	16
*mef*(A)	*mef*(A)_10_AF376746	Macrolide	13	0	0	0	0	0	0
*aadD*	*aadD*_1_AF181950	Aminoglycoside	10	0	0	0	0	0	0
*msr*(D)	*msr*(D)_2_AF274302	Macrolide, lincosamide and streptogramin B	7	0	0	0	0	0	0
*tet*(O)	*tet*(O)_2_M20925	Tetracycline	5	0	0	0	0	0	0
*tet*(O)	*tet*(O)_3_Y07780	Tetracycline	5	0	0	0	0	0	0
*mph*(C)	*mph*(C)_2_AF167161	Macrolide	3	0	0	0	0	0	0
*tet*(O)	*tet*(O)_1_M18896	Tetracycline	3	0	0	0	0	0	0
*mecA*	*mecA*_10_AB512767	β-lactam	2	0	0	0	0	0	0
*cfxA2*	*cfxA2*_1_AF504914	β-lactam	1	0	0	0	0	0	0
*cfxA*	*cfxA*_1_U38243	β-lactam	1	0	0	0	0	0	0
*msr*(A)	*msr*(A)_1_X52085	Macrolide, lincosamide and streptogramin B	1	0	0	0	0	0	0
*tet*(M)	*tet*(M)_3_U08812	Tetracycline	1	0	0	0	0	0	0
Total number of reads			847						

## Supplementary Materials

Supplementary materials can be found at www.mdpi.com/1422-0067/18/9/2011/s1.

## Abbreviations

BAL	Broncho-alveolar lavage
WMGS	Whole-metagenome shotgun
AST	Antibiotic susceptibility test
ARD	Antibiotic resistance determinant
