# Supplementary material for: Detection of Bacterial Pathogens from Broncho-Alveolar Lavage by Next-Generation Sequencing
Source: Int J Mol Sci. 2017 Sep 20;18(9):2011. doi: 10.3390/ijms18092011 (PMC5618659; doi:10.3390/ijms18092011)
Supplement: Supplementary file 1 [file ijms-18-02011-s001.pdf]

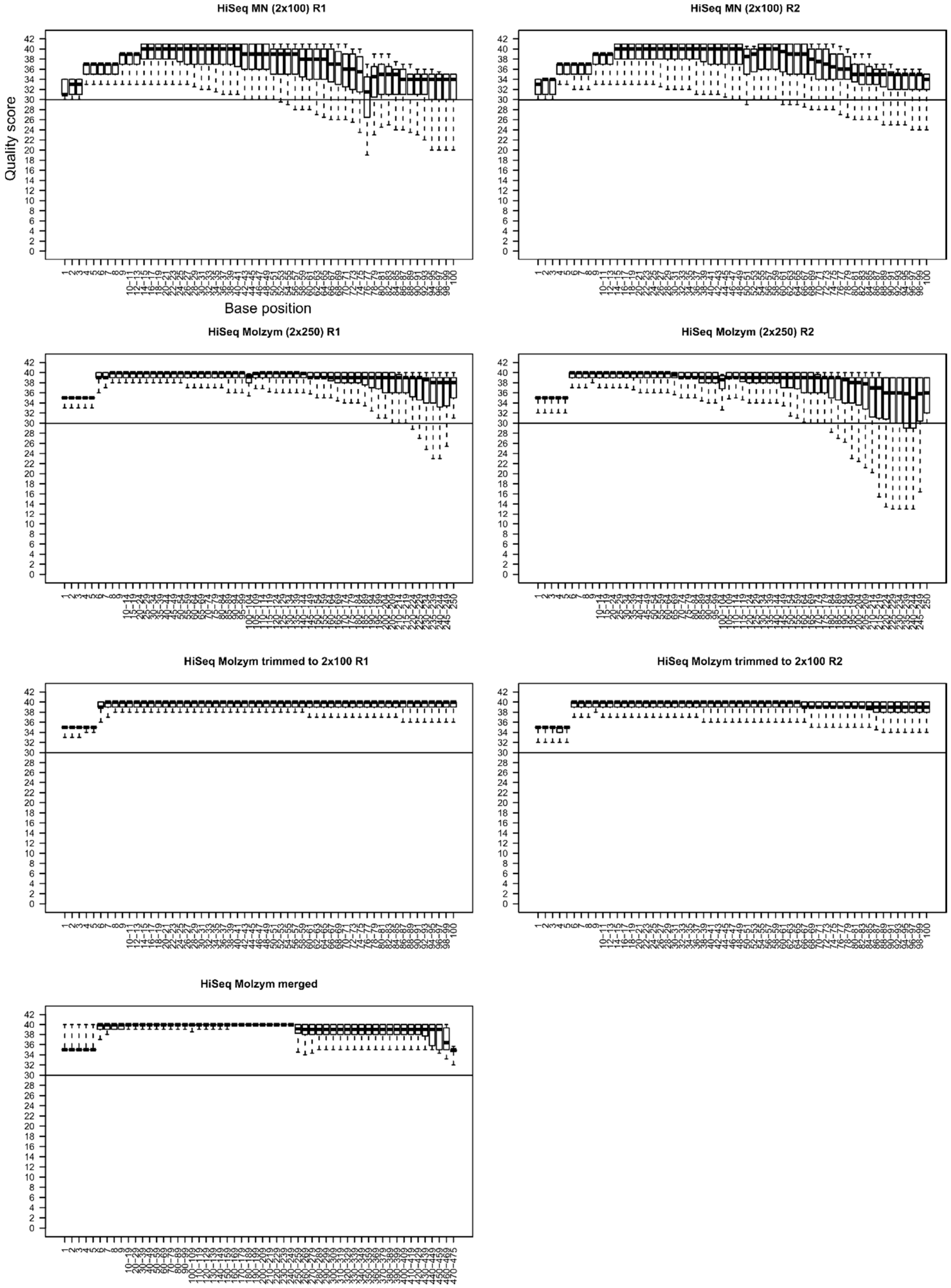

Figure S1. Boxplots of quality base score per base position obtained with the FastQC and re-plotted with R. Quality score represents the prediction of the probability of an error in base calling during sequencing where higher values correspond to more accurate detection of a specific base at a given position of a read. Each box and its "whiskers" show the distribution of quality score values; from the bottom to the top: the first, the second and third horizontal segments of each box represent the 10th percentile, 50th percentile or median and 95th percentile of quality score data values, respectively. R1 and R2 are forward and reverse reads, respectively. Horizontal continuous lines mark a quality score of 30 that corresponds to the error probability in base calling of 0.001.

Table S1. Clinical report of antibiotic susceptibility tests from the bacteriology laboratory for all the species identified by culture in the BAL sample [1].

**Class**	**Antibiotic**	***M. abscessus***	***C. jeikeium***
β-lactam	Meropenem	32 R	
	Imipenem	4 S	
	Penicillin		RES
Aminoglycosides	Amikacin	4 S	
	Gentamicin		RES
	Tobramycin	8 R	
Fluoroquinolone	Ciprofloxicin	2 I	RES
	Moxifloxacin	4 R	
	Levofloxicin	8 R	
Glycopeptide	Vancomycin		S
Glycylcycline	Tigecyclin	I	
Lincosamide	Clindamycin		RES
Macrolide	Clarythromycin	<0.5 S	
Oxazolidinone	Linezolid	8-16 I	
Tetracycline	Minocyclin	256 R	
	Tetracyclin		S
	Deoxycyclin	256 R	
	Ethambutol	64	

[1] I = intermediate susceptible; S = susceptible; R and RES = resistant; numbers indicate minimum inhibitory concentration (MIC) of the antibiotic expressed in mg/L. The antibiogram is interpreted according to the guidelines of the European Committee on Antimicrobial Susceptibility Testing (EUCAST, edited in 2014).

Table S2. Whole-genome sequencing depth for *C. jeikeium* and *M. abscessus* [1].

	***C. jeikeium* K411**		***M. abscessus* ATCC 19977**	
**Reference genome size (nt)**	2462499		5067172	
**Statistics**	**CLARK**	**Kraken**	**CLARK**	**Kraken**
# of reads [2]	779742	834581	338846	341917
Mean depth per position [3]	91.614	98.674	21.256	21.440
Median depth per position [3]	25	35	21	21
Genome coverage (%) [4]:				
1x	58.347	59.284	97.818	97.821
5x	54.440	55.797	96.496	96.527
10x	52.871	54.343	89.210	89.391
15x	51.727	53.269	72.360	72.900
20x	50.756	52.388	50.111	50.850
30x	48.893	50.685	15.747	16.324
50x	45.897	47.875	0.458	0.501
100x	38.969	41.540	0.001	0.010

[1] Sequences classified by CLARK or Kraken to *C. jeikeium* or *M. abscessus* were extracted from quality-filtered and merged read pairs dataset of the enriched BAL sample. Eventually, mapping against reference genomes was performed with BWA with default settings. Read depth was analyzed with SAMtools and computed with R.; [2] Total number of reads assigned by CLARK or Kraken to a given reference genome; [3] Mean and median number of reads per position; [4] Percentages of bases of the reference genome covered at different read depth (e.g., 1x = one read depth).
